# Climate change and non-communicable diseases: An invisible syndemic

**DOI:** 10.1371/journal.pmed.1005082

**Published:** 2026-05-08

**Authors:** Gokul Parameswaran, Sadeer Al-Kindi, Sanjay Rajagopalan

**Affiliations:** 1 Cardiovascular Research Institute, Case Western Reserve School of Medicine, Cleveland, Ohio, United States of America; 2 Harrington Heart and Vascular Institute, University Hospitals, Cleveland, Ohio, United States of America; 3 Division of Cardiovascular Prevention and Wellness, Houston Methodist, Houston, Texas, United States of America

## Abstract

In this Perspective, Sanjay Rajagopalan and colleagues discusses how climate change accelerates non-communicable diseases (NCDs) and exacerbates NCD-related mortality, and calls for greater visibility and funding to address this syndemic and improve human health.

## Teaser

Climate change accelerates non-communicable diseases (NCDs) through cascading environmental disruptions and is attributed to driving increased NCD-related mortality. Yet this syndemic remains invisible and underfunded. We detail why addressing the climate-NCD intersection is critical for improving health.

Disruption of multiple planetary boundaries due to anthropogenic activities, primarily related to fossil fuel combustion, profoundly threatens human health, with over 3.6 billion people living in climate-vulnerable regions [1]. In 2024, Earth’s temperature crossed the critical threshold of 1.5 °C above preindustrial levels, and with current policies the world is heading towards a 3 °C rise by 2100 [2]. This will have significant impacts on non-communicable diseases (NCDs) such as cardiovascular disease (CVD) and cancer—which account for 38 million deaths annually—as NCDs can be driven or exacerbated by environmental stressors from planetary system changes [3].

Climate change acts as an NCD “syndemic” accelerator by intensifying and synchronizing existing disease with social and environmental vulnerabilities, amplifying combined impact beyond the sum of their parts [4]. Many primordial risk factors linked to NCDs (e.g., obesity, hypertension, and lack of sleep) may themselves be shaped by these disrupted environments [1,3]. However, NCDs lack the visibility of infectious disease (ID) epidemics, with no sudden clusters, clear pathogens, or transmission risk. Most deaths are never attributed to environmental exposures as causal chains are complex, non-linear, and delayed, resulting in a causal attribution gap rendering the climate-NCD syndemic comparatively invisible, despite dwarfing ID mortality. In this Perspective, we detail why the climate-NCD intersection should be a priority for impactful intervention.

## Planetary disruption and disease: An integrated framework

**Fig 1:**
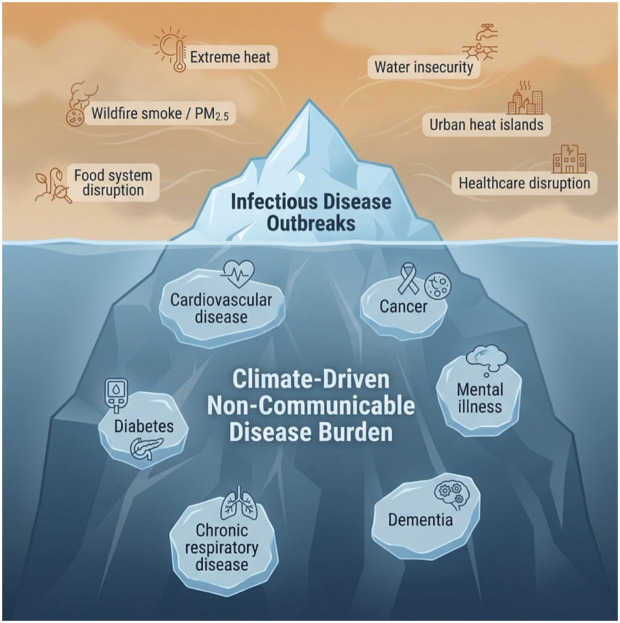
The climate-NCD syndemic iceberg: Hidden dimensions of climate-driven disease burden. Climate change generates a constellation of environmental stressors—including extreme heat, wildfire smoke, water insecurity, food system disruption, urban heat islands, and healthcare disruption—that drive the visible burden of infectious disease outbreaks. Beneath the surface lies a far larger, largely invisible syndemic of climate-driven non-communicable diseases, encompassing cardiovascular disease, cancer, diabetes, chronic respiratory disease, dementia, and mental illness. Figure created using Nano Banana AI.

Climate change generates multiscale disruptions across different planetary systems—the atmosphere, hydrosphere, pedosphere, cryosphere, biosphere, geosphere, and anthroposphere—that create interacting, cascading, and compounding exposures (Fig 1). The effects of disruptions on NCDs can be both direct and indirect and may operate across varying time scales. The most established direct pathway operates through atmospheric warming. Heat, an independent cardiometabolic stressor, has driven a 63% rise in heat-related mortality since the 1990s, exceeding 546,000 deaths annually [2]. Warming simultaneously degrades air quality by accelerating pollutant formation, hindering pollutant dispersal, and creating drier conditions that intensify wildfires and dust storms [5]. Together, these exposures interact to produce multiplicative increases in mortality, driving multi-organ NCD burden across cardiovascular, respiratory, and neurological systems [5]. In one of the first studies to causally link climate change with wildfire smoke and premature mortality, warming climate conditions are projected to cause ~71,000 excess annual deaths and nearly 1.9 million cumulative excess deaths between 2026–2055 [6]. Beyond physical health, increasing frequency of extreme weather events worsens mental health by increasing mood disorders and trauma-related illness [7]. Indirect pathways, meanwhile, operate primarily through disruption of the systemic infrastructure protecting health. Hydrosphere and pedosphere disruptions characterized by altered precipitation, drought, and soil degradation erode freshwater availability and nutritional quality. The resulting climate-related shifts in agricultural output risk increasing reliance on calorie-dense ultra-processed foods, accelerating metabolic disease, and potentially driving over 500,000 deaths annually by 2050, with many of these attributable to CVD and cancer [8,9]. Cryosphere changes, including glacial melt and sea level rises, may result in population displacement, severing communities from stable food and healthcare access. These pressures converge on the anthroposphere with urban systems, healthcare infrastructure, and supply chains buckling under compounding climate stressors. Behavioral adaptations compound these risks, as hostile temperatures may discourage physical activity and increase harmful indoor exposures, such as fungal spores from mould or volatile organic compounds from paints [10]. Collectively, these interacting disruptions transform climate change from a singular threat into a syndemic driver, overwhelming public health models focused on isolated risk factors than integrated planetary-scale exposures.

## Climate vulnerability: Disproportionate burdens and cumulative disadvantage

Climate change disproportionately burdens socioeconomically disadvantaged populations, constituting a fundamental equity crisis. Most Global South countries sit at the geographic fault lines of climate disaster, where rising sea levels, shifting weather patterns, and extreme events cascade across ecosystems to expose systemic vulnerabilities [1]. Reliance on climate-sensitive sectors, fragile infrastructure, and chronically underfunded health systems limits capacity to respond to disasters or adapt to shifting planetary systems [1]. These asymmetries translate climate shocks into compounding NCD burdens across already vulnerable regions of the planet, despite historically minimal emissions. In the U.S., formerly redlined neighborhoods—systematically denied investment based on race or ethnicity—often feature dense impervious surfaces, limited tree canopy, aging housing, and proximity to major roadways, amplifying pollution exposures and fostering obesogenic environments [11]. Occupational exposures further compound risk, as lower-income communities predominantly work outdoor or manual labor jobs with high heat exposure [5]. National analyses have shown higher climate vulnerability measures, integrating cumulative environmental stressors, pollution, and adaptive capacity, are associated with greater risk of cardiometabolic disease, independent of other baseline social and infrastructural features [11].

## Why we fail to act: Barriers to prevention

Despite mounting evidence linking climate change to NCD mortality, there is a large gap in implementing adaptation and mitigation strategies. NCDs operate through diffuse and delayed pathways obscuring causation and enabling political gridlock. Partisan polarization has led to trust deficits fueling climate denialism and obstructing interventions. Recent U.S. decisions exemplify this: withdrawal from the Paris accord, rollback of environmental regulations, reduced authority of the Environmental Protection Agency, and halting renewable investments have reversed American decarbonization efforts [12]. Policy resistance arises from misalignment between short-term political horizons and long latency of environmental exposures. Individuals often underestimate environmental risks given the protracted development of NCDs and near invisibility of these exposures. Unlike pandemic preparedness, which commands emergency appropriations, climate-NCD prevention receives no comparable funding, despite vastly exceeding ID mortality. Economic barriers further impede action, as adaptation requires substantial upfront investment, including retrofitting power/transportation networks, building climate-resilient infrastructure, or providing community education, which only gradually accrue benefits. This mismatch discourages long-term political commitment, especially when climate adaptation competes with more immediate priorities.

## Towards a multi-level adaptation strategy

Current and projected climate impacts demand urgent adaptation strategies within communities, environmental infrastructure, and healthcare systems [3]. Community interventions must include targeted education for vulnerable populations, including teaching practical avoidance strategies at peak exposures. In addition, subsidizing adaptive technologies like air conditioning or air purifiers for high-risk households or communities may prove cost-effective. Infrastructure interventions will require interdisciplinary collaboration, such as healthcare professionals engaging with urban planners in designing climate-resilient environments through increased vegetation, shaded pathways, and reflective roofing. Early-warning systems integrated with public cooling/clean-air centers would also provide critical refuge during extreme events. In healthcare settings, environmental risk assessments should be incorporated into patient counseling, especially for vulnerable populations on drugs affecting thermoregulation [5]. Healthcare systems must also strengthen disaster preparedness through climate-resilient infrastructure, ensuring supply chain continuity and expanding decentralized infrastructure via mobile clinics to maintain access during emergencies [5]. Reframing climate change as a health imperative makes visible its direct role in shaping the global burden of NCDs, and anchors climate action within clinical, public health, and policy decision-making. Because climate harms are unequally distributed, only an equity-centered approach that meaningfully engages the Global South and structurally disadvantaged populations may generate durable solutions [1].

## Conclusions

The climate–NCD syndemic is likely the defining public health issue of the 21st century. Meeting this challenge will require not only innovation, but ethical leadership and political courage to confront entrenched systems that sustain delay. Anchoring climate action in the universal imperative of health may be the clearest pathway to bridging ideological divisions and catalyzing sustained collective action.

## Abbreviations

CVD: cardiovascular disease
ID: infectious disease
NCDs: non-communicable diseases
